# Community perspectives on dogs, health risks, and veterinary care impacts in rural Alaska

**DOI:** 10.3389/fvets.2025.1602564

**Published:** 2025-07-23

**Authors:** Laurie Meythaler-Mullins, Sama Johnson, Allyce Lobdell, Claire Hood, Claire Kazen, Danielle M. Frey

**Affiliations:** College of Veterinary Medicine and Biomedical Sciences, Colorado State University, Colorado, CO, United States

**Keywords:** one health, Alaska Native, dogs, access to veterinary care, rabies, public health, rural, Alaska

## Abstract

Veterinary care in much of Alaska is extremely limited due to the high cost of living and low access to care. To date, community perspectives on veterinary care and the efficacy of veterinary services introduced have not been documented in Alaska Native communities, in this case specifically the Yukon Kuskokwim (YK) Delta of Alaska. This stymies the introduction of thoughtful public policy created with community insights. It also impedes program funding and awareness of the public health importance of veterinary care. This study aims to utilize information from surveys to form a basis in literature for veterinary medicine in rural Alaska and to document community perspective input to aid in making veterinary care sustainable in this region. Using information gathered from 390 surveys completed in 25 YK Delta communities between 2019–2024, The authors found general support for dogs being a part of the community and culture in YK Delta communities. Specifically, nearly all respondents like dogs (95%), 94% of respondents reported owning animals, and dogs are the most common type of animal owned or cared for in this region by far (92%). Yet community members feel that stray and unwanted dogs are a problem (71%) and loose dogs are feared (69%). Respondents also report that dogs spread waste and garbage around their community (75%). Perceptions of and actual ability to access veterinary care are low. Community members report dogs have low rates of vaccination (a reported 62% rate of rabies vaccination) and low sterilization rates (53% reported rate of sterilization surgeries completed). Consequences of overpopulation noted by respondents also include dog bites and attacks, and negative impacts of having to remove unwanted dogs from the community. Recommendations are given on actions to take on the federal, state, city, and village levels to increase access to veterinary care based on these findings. Increasing awareness of the current state of veterinary care and the potential benefits to humans and animals is a key step to improving access to care.

## 1 Introduction and background

The human-dog connection is an important cultural aspect of life in Indigenous communities in Alaska and has been for centuries. Dogs have been crucial not only for companionship but also for transportation, subsistence, and protection. Over the past two centuries, colonization has changed Native Alaskan relationships with dogs. For example, the invention of the snow machine limited the function of dogs in daily life. The long-term effects of these changes have led to overpopulation and high numbers of free-roaming dogs. This shift in use and population leads to critical questions about how these communities value and perceive dogs now. This knowledge may specifically inform the creation of healthcare and population management processes with dogs and community health in mind.

In Alaska, dog overpopulation, combined with the primarily free-roaming dog population, is a significant health threat to communities. This is due to increased incidences of dog bites and a higher risk of zoonotic disease transmission (1). This paper focuses specifically on the realities of life in Southwest Alaska, a region known as the Yukon Kuskokwim Delta (YK Delta), as it pertains to community perspectives on attitudes toward dogs, health risks associated with them, and accessibility of veterinary care.

The public health challenges of dog bites, rabies exposure, and other disease exposures are daily realities for this region. The primary regional zoonotic disease concern is rabies, which is endemic in the local fox population in the YK Delta. This increases the risk of rabies exposure in village residents through contact with loose, unvaccinated dogs that may have come in contact with rabid wildlife. While the human healthcare system in the rural regions of Alaska is well-established and able to support humans in response to these bites and zoonotic diseases, there is no system to provide veterinary care in remote, sparsely populated areas. Without accessible veterinary care to address the preventable aspects of these diseases, there exist major challenges for public health in large areas of Alaska.

To date, community perspectives on veterinary care and the efficacy of veterinary services introduced have not been documented in Alaska Native communities. This stymies the introduction of thoughtful public policy created with community insights. It also impedes program funding and awareness of the public health importance of veterinary care. As the health of dogs is closely tied to the health of humans, the ability to improve access to veterinary care through these mechanisms may result in improved health outcomes for community members.

This study uses information gathered from surveys collected by a team of veterinarians from Colorado State University (CSU) and the University of Alaska Fairbanks (UAF) as part of the Hub Outpost Program (HOP) while delivering preventive veterinary care in the region over 5 years. This paper describes and discusses the results of this survey completed by residents of the YK Delta region of Alaska. It assesses community perspectives on dogs, health, and accessible veterinary care. Specifically, the purpose of this study is to assess and describe: (1) personal and community attitudes toward dogs; (2) perceptions of access to veterinary care and dog-related health problems; and (3) perceived outcomes of the HOP presence in the community. As a first undertaking in measuring this region's community attitudes, these results can be used for developing policy, programs, and educational campaigns, ideally resulting in improved health outcomes for community members.

### 1.1 Alaska geography and culture

**Figure 1:**
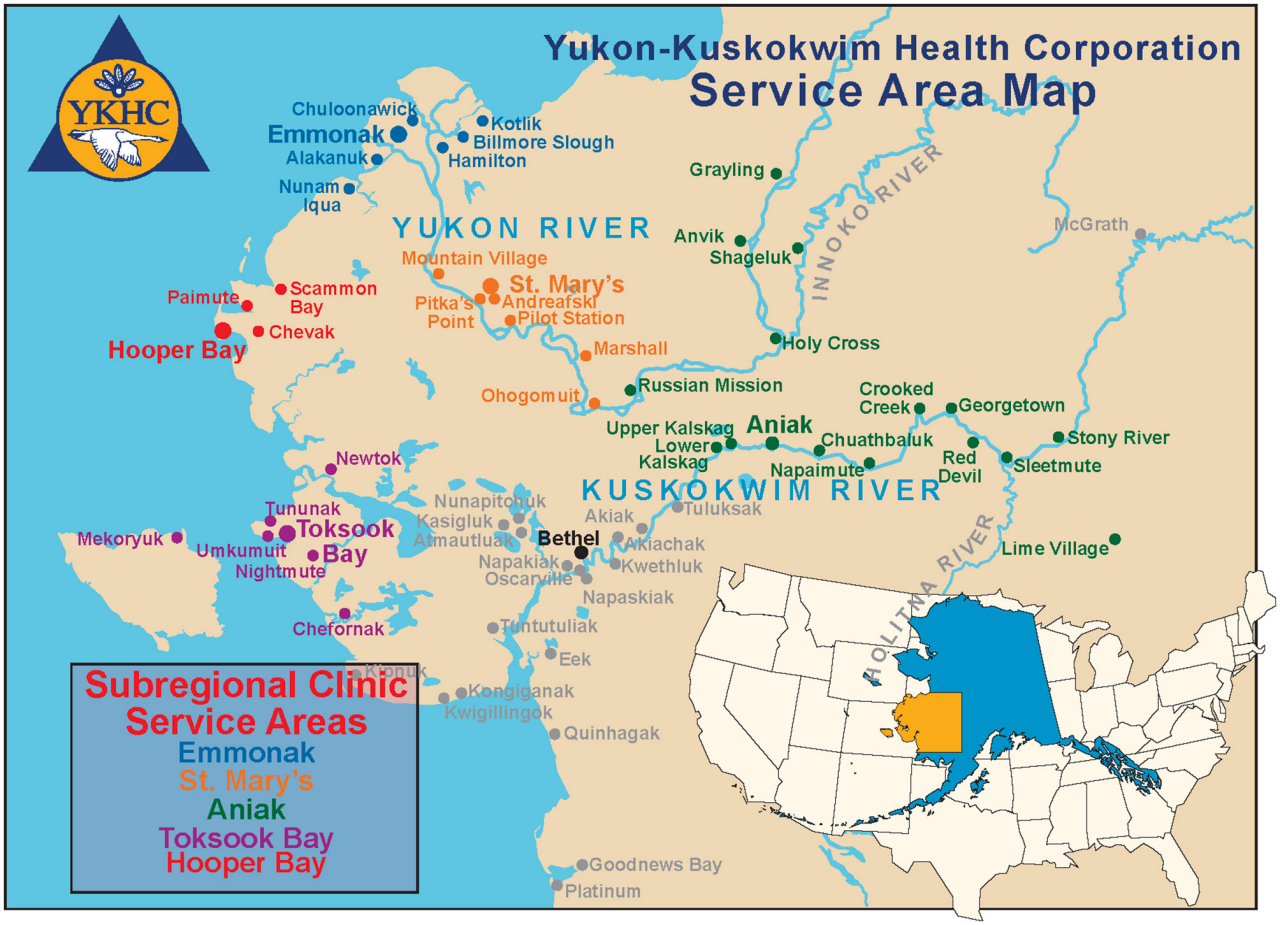
Yukon Kuskokwim health corporation service map.

To understand the perspectives shared and the impact of the responses, it is critical to understand the geography, culture, and reality of dogs in the region. Alaska is the largest state in the U.S. (Figure 1) (2). Despite its size, Alaska has only 17,637 miles of public roads. As a comparison, Texas, the second-largest U.S. state by area, has eighteen times as many miles. Air travel is the primary method of transportation throughout much of Alaska. This is especially true for travel into and out of Alaska Native villages, as most are not connected to the state's limited road system.

The YK Delta region of Alaska, the regional focus of this paper, occupies the southwest portion of the state (Figure 1). This region, with an area of 75,000 square miles, is approximately the same area as the US state of Oregon. It has a population of just over 23,000 people who live across 58 rural communities, each home to a federally recognized Tribe. These communities vary in population from 25 to over 1,000 people. Bethel is the regional service and transportation center with a population of nearly 7,000 people. The Bethel Airport provides daily flight service to the larger cities of Anchorage and Fairbanks as well as to villages throughout the region. From the villages, Bethel is only accessible by airplane, boat (in warmer months), or snowmobile and four-wheeler (in the winter).

This region is home to three cultures: Yup'ik, Cup'ik, and Athabascan. The Yup'ik have lived throughout the region traditionally; the Cup'ik called Nunivak Island and coastal villages home; and the Athabascans have traditionally resided along the Yukon River and interior villages. While food and supplies are delivered to the villages by air freight, many Alaska Natives in the region still rely on a subsistence lifestyle due to the costs of food.

### 1.2 Veterinary Care in Alaska and HOP

Veterinary care in much of Alaska is extremely limited due to the high cost of living and low access to care due to socioeconomic and geographic barriers. Most rural Alaska communities do not have local veterinarian practitioners and are too small to support a clinic on their own without some form of outside support. Therefore, residents must travel to or wait for one to visit. The costs for veterinarians associated with travel to the communities make it economically unsustainable for practices to provide continued service to these communities. Thus, access to veterinary services is typically limited to those who can afford to fly their animal to a larger city (at a cost ranging from hundreds to thousands of dollars). This is unaffordable to most dog owners in this region. Exacerbating the shortage of available veterinary services, high veterinary costs impose additional barriers to accessing preventative care for animals. Of the communities visited by HOP, Bethel is distinct in its access to veterinary care in that it has a veterinarian that provides services in town 1 week per month. Bethel residents also have access to a direct flight to Anchorage where they can transport their animal to receive care.

Based on this information, it is clear there is a critical need to find solutions for accessible veterinary care. Consistent low-cost or no-cost community-based programs offer one potential solution. However, careful attention to community communication, empathy, and cultural sensitivity are critical in ensuring the success of such programs (2, 3). These tenants are necessary for programs to create a positive foundation for future veterinary care and an ongoing relationship between pet owners, their pets, and veterinary professionals (4). Work in Northern Canada, a region with similar geography and indigenous populations as the YK Delta, documents programs that exhibits these tenants and simultaneously improved access to veterinary care as well as decreased dog populations (5, 6).

Community perspectives and input into the development of these programs are essential. In Canadian Indigenous communities challenged by canine overpopulation, community members view current management practices that lack community input and support as unacceptable (7). On the other hand, programs formed with community needs integrated from the beginning have more local support and are projected to have better long-term outcomes (3, 5, 8).

The Hub Outreach Program (HOP) was initiated in part to address this lack of accessibility in the YK Delta of Alaska with input from the Yukon Kuskokwim Healthcare Corporation (YKHC), a Tribal Organization that is authorized by the tribal governing councils to work with Indian Health Services to provide human healthcare in this region. HOP was developed specifically to use a One Health framework, a perspective that focuses on the interconnectedness of humans, animals, and the environment, to find pathways for sustainable veterinary care and address public health threats through preventative veterinary medicine (9). HOP was first envisioned in 2017, and the regional partnership with YKHC was developed in 2018. The program began delivering preventative, public health veterinary services to the YK Delta in 2019. The HOP model was designed based on the YKHC human healthcare delivery model. Using a hub-and-spoke design model, the program deploys a veterinary team to strategically identify communities from the “hub” of Bethel. Veterinary field clinics are established in outlying “spoke” communities. Each “spoke” community is accessible to an additional five to ten communities.

### 1.3 Dogs and health risks

In the communities served by the HOP, there are high numbers of dogs. Out of the 4,884 animals treated during HOP veterinary clinics, 4,700 or 96.2% of the animals presented were dogs and 184 animals or 3.8% were cats.

Culturally, dogs have been crucial in this region not only for companionship but also for transportation, subsistence living, and protection. Through the twentieth century, sled dogs were the most common means of transportation for nomadic communities in the circumpolar north. Dogs have assisted with protection and hunting as well, playing a critical role in the subsistence lifestyle common in Alaska both historically and contemporarily. However, at the end of the twentieth century, forced settlement and colonial implementation of Western education and health care led to cultural shifts, including impacts on the human-dog connection. There was a decreased reliance on dogs for work as the previously nomadic population became settled and concentrated. The transition to snow machines for transportation further decreased this reliance. Along with people, dogs became settled and concentrated in a small area. When dogs were no longer working, their heat and breeding cycles were not monitored. A female dog can have up to 2 litters of puppies per year with as many as 12 puppies per litter. Depending on her size, a female dog will begin cycling around 6 months of age. As a result, many remote communities have issues with dog overpopulation (10). These changes eventually resulted in dog overpopulation.

Contemporarity, dogs in YK Delta can be divided into two main groups *owned* and *unowned*. The owned dog population is dependent upon humans for food, water, and shelter and includes dogs who are kept in a limited area (e.g., in a dog yard), and those that are free to roam without human supervision, or *loose*, as the locals refer to them and as were asked about in the survey questions. A single dog may have one owner or may be a *community dog*, having more than one owner. Unowned dogs (often referred to as *stray*) do not have an owner but may still depend upon humans directly or indirectly for food, water, and shelter.

Understanding this loose dog population is particularly important to understanding public health issues and impacts. The current dog overpopulation combined with the primarily free-roaming population and the lack of veterinary care pose a significant health threat to Alaskan communities due to increased incidences of bites and a higher risk of zoonotic disease transmission. Rabies prevalence is of unique importance in this region. Hospitalization rates from dog bites are higher in Alaska compared to the average rate in the United States with the Alaska Native population being disproportionately affected. Furthermore, dog bites are a significant cause of injury in Alaska Native children with higher rates compared to the general United States child population (11).

With a high prevalence of dogs and a lack of access to veterinary care, unvaccinated dogs may encounter rabid wildlife and then bring the disease into contact with the humans who care for them. Since rabies is a fatal virus, any possible human exposure to rabies results in lengthy rabies post-exposure vaccine series and treatments. In 2021, for example, three rabid foxes were identified in three different villages in the YK Delta. These rabid foxes had known contact with unvaccinated owned dogs, and the dogs had to be euthanized. In these cases, the potentially exposed owners were required to travel by plane to Bethel for treatment and missed work for up to 2 weeks to receive the care that was needed. This event demonstrates the toll that a lack of veterinary care can have on the individuals involved and the Indigenous community, including negative impacts on mental and physical health, lost revenue, out-of-pocket expenses, and lost manpower in the community.

## 2 Materials and methods

This study is cross-sectional, utilizing data from a survey titled “Dogs in My Community” collected from residents of the YK Delta in Alaska. The survey was developed by HOP leaders to evaluate community and personal attitudes about dogs in the YK Delta. The survey was disseminated in rural Alaskan villages visited by HOP between 2019 and 2024. Data was then compiled and assessed by the HOP team.

### 2.1 Survey design

The survey was written into PDF format based on anticipated in-person survey dissemination and includes 42 questions divided into five sections (see Appendix A). Topics include canine interaction and community role, impacts on human health, perception of veterinary care, access to veterinary care, and canine demographics. Questions and response options are provided here, with many response options in parentheses following description of the question.

In the first survey section there were five questions around general perceptions of dogs. First, respondents were asked how they feel about dogs with response options including “I like dogs,” “I am indifferent to dogs,” “I don't like dogs,” and “I am afraid of dogs.” They are also asked about what their primary interaction with dogs is with the question “How do you interact with dogs” and response options of “I own pet dog(s),” “I work with dog(s),” and “I do not interact with dogs.” The next question asks respondents about the nature of those interactions with the question “My ineraction with dogs is” followed by three response options of “positive,” “neutral,” and “negative.” Next, respondents are asked if they own animals by circling either yes or no. If they circled yes, they were asked how many animals they own by providing a line after the text to allow respondents to write in a number. Also if the respondents did indicate owning animals, they were saked “What types of animals do you own? Dogs, Cats, Other ______.” If respondents answered that they do not own any animals, they were asked why not with an open response option to write in an answer. The last question in this section asks respondents “What role do you think dogs play in your community?” with an open response option to write in an answer.

The second section of the survey asks questions on veterinary care (questions 6–17). First, in question 6 of the survey respondents are asked if they have heard of the veterinary profession before (yes/no) and if not, did they know what a veterinarian does (yes/no). In question 7 respondents were asked if a veterinarian has ever visited their community (yes/no/I don't know) and if so, when the last time a visit occurred (open text). The eighth question asks if the respondent thinks they are able to access veterinary care (yes/no) and if not, why (open text). Question nine asks if the respondent has ever accessed veterinary care for their animal (yes/no). If yes, respondents were asked to identify the mechanism they used with four response options: a) “I called a veterinarian or veterinary technician for advice,” b) “Me and/or my animal traveled outside of my community to visit a veterinarian,” c) “A veterinarian or veterinary group visited my community,” or d) “A non-veterinary group helped me access veterinary care.” If they had not ever accessed care for their animal, they were asked why not (open text). Question 10 asks if respondents think veterinary care is affordable (yes/no/I don't know).

The next set of questions within this section asked respondents about specific veterinary care. Respondents were asked if any of their dogs have been dewormed in question 11 (yes/no/I don't know) followed by question 12 which asks “When was the most recent deworming for any of your dogs?” Response options for question 12 include (a) “ <1 month ago,” “ <1 year ago,” “1–2 years ago,” “3–5 years ago,” “>5 years ago,” and “I don't know.” Similarly, respondents were asked if any of their dogs had been vaccinated for rabies (question 13), and if so, when the most recent vaccination for rabies had occurred for any of their dogs (question 14) with identical response options as question 12. Question 15 asks respondents if any of their dogs have had any other vaccinations aside from rabies (yes/no/I don't know) and question 16 asks how recently this occurred with identical time interval response options to questions 14 and 12. The last question in this section asks, if they own dogs, if respondents' dogs are spayed or neutered (yes/no) followed by open text for why or why not they have spayed or neutered them.

The third section includes questions 18–33 which asks questions related to broader community views of dogs. The first five questions are on how dogs exist in the communities. Starting with question 18, “How do you identify a dog as being owned or unowned” with an open text response option. Question 19 asks if dogs typically have identification (yes/no/I don't know) followed by question 20 on if dogs are usually fenced/tied when outdoors (yes/no/I don't know). The next two questions (12, 13) have open text response options, first asking how many “loose” dogs respondents saw per day then asking how many “un-owned” dogs saw per day.

The next set of questions in this section ask about the respondents' views on dogs in their communities. Question 23 asks respondents if “stray or unwanted” dogs are a problem in their community (yes/no/I don't know). Next, question 24 asks if people in the respondent's community fear loose dogs (yes/no/I don't know). Then respondents are asked how safe they feel being around dogs with response options of “not at all safe,” “indifferent,” and “very safe.” Question 26 asks if respondets are less will to exercise outside because of dogs (yes/no/I don't know).

The next set of questions in this section ask about the connect between dogs and humans in the community. Question 27 asks if dogs spread waste or garbage around the respondent's community (yes/no/I don't know). Then respondents are asked if they think dogs in their community make people sick (yes/no/I don't know). “Have you ever been treated for a disease that you may have gotten from your dog?” with response options of yes, no, and I don't know is question 29. If yes, respondents were asked to choose from a list of diseases/issues including (a) ringworm, (b) GI problems, (c) cysts in your chest or abdomen, and (d) other with open text to write in an answer.

The final set of questions in this section revolve around dog bites and attacks. Question 30 asks if dog bites or attacks are a problem in the respondent's community (yes/no/I don't know). In question 31, respondents are asked if they own a dog, has it ever been bitten by another dog or wild animal (yes/no/I don't know/I don't own a dog). Respondents were then asked if they would report if their dog had been bitten by another dog or wild animal (yes/no/I don't know). Respondents were asked to explain why or why not. Finally, in question 33 respondents were asked if they had ever been bitten by a dog (yes/no/I don't know) and if so, if they reported it. This question concludes with asking respondents to explain why they did or did not report the bite.

The fourth section of the survey investigates the cost of dog-related health problems with five questions (34–38). Question 34 asks if the respondent have ever received medical treatment for a health issue resulting from exposure to a dog (yes/no/I don't know). Next, respondents are asked if they have ever received medical treatment for a dog bite (yes/no). If yes, they are asked if they had to leave their community to receive treatment (yes/no) and if they did, how much money did they have to spend on travel, lodging, and meals while away (open text). Within this question respondents were asked if there were medical costs that insurance did not cover (yes/no) and how much (open text). Next, respondents were asked if they lost wages or the ability to work due to a dog bite (yes/no) and if yes, how much money was lost (open text). Respondents were then asked how long they were unable to work or carry out normal daily activities (open text).

Questions 37 and 38 revolved around dealing with unwanted dogs in the community. Question 37 asked respondets how they handle unwanted dogs. Respondents were allowed to pick more than one option from the following: (a) “send them out to rescue groups,” (b) “My community takes care of this [organized, scheduled and/or announced community wide culls (kill days)],” and (c) “dog owners have to take care of this themselves.” Finally, question 38 asks if respondents have to put down dogs regularly, do it negatively affect them (yes/no/I don't know).

The survey concludes with a short four-question section on the perceived outcomes of the Hub Outpost Program in the community. Question 39 asks if the respondent heard about the program before it visited their community (yes/no) and if so, ohw they heard. Response options to the latter include (a) radia, (b) facebook, (c) word of mouth, and (d) “other” with open text to write in an answer. Question 40 asked if the respondent noticed any changes in their community since the program began (yes/no/I don't know) and if so, what those changes are. In question 41, respondents are asked if their view of dogs in the community has changed since the program began (yes/no/I don't know) and to explain in open text. The last question of the survey asks respondents to write in anything else they would like to share in open text.

### 2.2 Study sampling

Sampling for this study occurred in rural villages in the YK Delta. Recruitment occurred at locations where the HOP hosted clincs in the region. HOP began operating in the region in 2019, marking the beginning of the survey period, and concluded survey gathering in 2024 as the program was coming to an end. In this time, HOP worked in twenty-five YK Delta communities offering subsidized services at no cost to pet owners, creating a convenient access point for recruiting residents for participation. Surveys were available on-site to owners who brought animals to HOP clinics and could complete the survey on-site. The sample extended to anyone in the visited communities, even if the participants did not visit a clinic. Surveys were also mailed to tribal councils, community leaders, and city employees who then distributed them within their respective communities. Surveys were then returned by mail. Importantly, owning an animal was also not required to participate in the study.

Surveys from all twenty-five communities were received, with a total of 390 surveys collected. The city of Bethel contributed nearly a quarter of all survey responses (23%). The final sample size was 390. These surveys were received from all twenty-five locations visit by HOP: Bethel (23%), Aniak (12%), Hooper Bay (12%), Quinhagak (8%), St. Mary's (8%), Akiak (6%), Tununak (6%), Chevak (3%), Emmonak (3%), Grayling (3%), Kalskag (3%), Akiachak (3%), Napaskiak (3%), Kwethluk (2%), Goodnews Bay (1%), Holy Cross (1%), Crooked Creek (1%), Kipnuk (1%), Nunapitchuk (<1%), Kongiganak (<1%), Toksook Bay (<1%), Chuathbuluk (<1%), Kasigluk (<1%), Oscarville (<1%).

### 2.3 Analyses

The survey contains primarily two types of question formats: multiple choice response options and open text response options. Therefore both quantitative and qualitative analysis methods were utilized in the analysis of the data. As an exploratory study of the conditions in rural Alaska, the analysis consisted of primarily descriptive statistics. The quantitative analyses were conducted with the statistical software R. Data was cleaned and appropriately coded for the intended analyses. Some data was more difficult to code for analysis. For example, respondents were asked how many loose dogs, on average, they see per day in their community with an open text response option. Many respondents provided ranges (e.g., 51–5 dogs), but others provided ranges without definitive quantities (e.g., 5 or more) and some respondents reported non-quantities such as “lots” or “too many to count.” Therefore, first the quantitative data were transformed into two variables of range minimums and maximums and then assessed in a frequency table (Table 3) with bins increasing by 10 (i.e., 1–10, 11–20, 21–30, etc.). For those ranges without an upper quantitative limit, no data were included. The same was done for the question asking about un-owned dogs seen per day. Once cleaning and coding was complete, frequency tables were run on all multiple choice data.

A thematic analysis was used on the open text data completed in Excel as other qualitative analysis software was not available to the research team. This analysis was conducted by identifying themes of, then coding, each response. Each questions' data went through at least 2 iterations of coding to find commonalities and distinctions. Then, each set of themes was counted and presented in a more quantitative approach using frequencies.

## 3 Results

Selected results of the mixed methods analysis are reported below. While results are reported for all locations for all topics, due to the high response from Bethel and unique nature of access to veterinary care in the city, analysis of specific questions without residents has also been included. This is further explained in the results section.

### 3.1 Personal attitudes toward dogs

**Figure 2:**
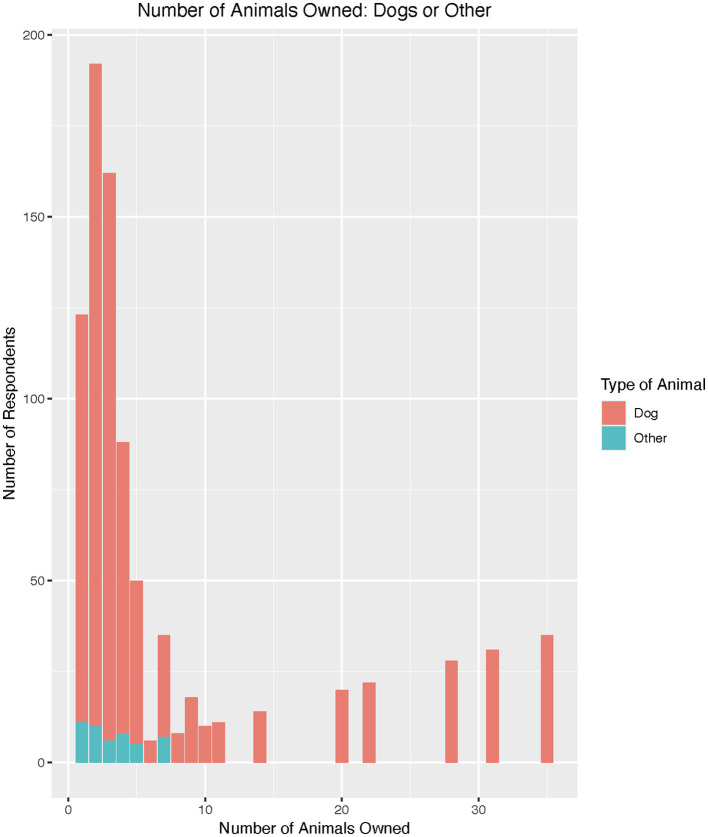
Reported numbers of animals owned by survey respondents.

Most respondents owned one animal (40%). About 28% of respondents indicated owning two animals, and 16% of respondents owned three animals. It was uncommon to own more than five animals, though several respondents reported owning much higher numbers, up to 50 animals (it is likely these are dog teams, as the sport of dog mushing is common regionally). Dogs are the most common animal owned or cared for in this region by far (92%; Figure 2). Cats were the next most common (14%). “Other” animals were indicated (3%) but not further described.

**Figure 3:**
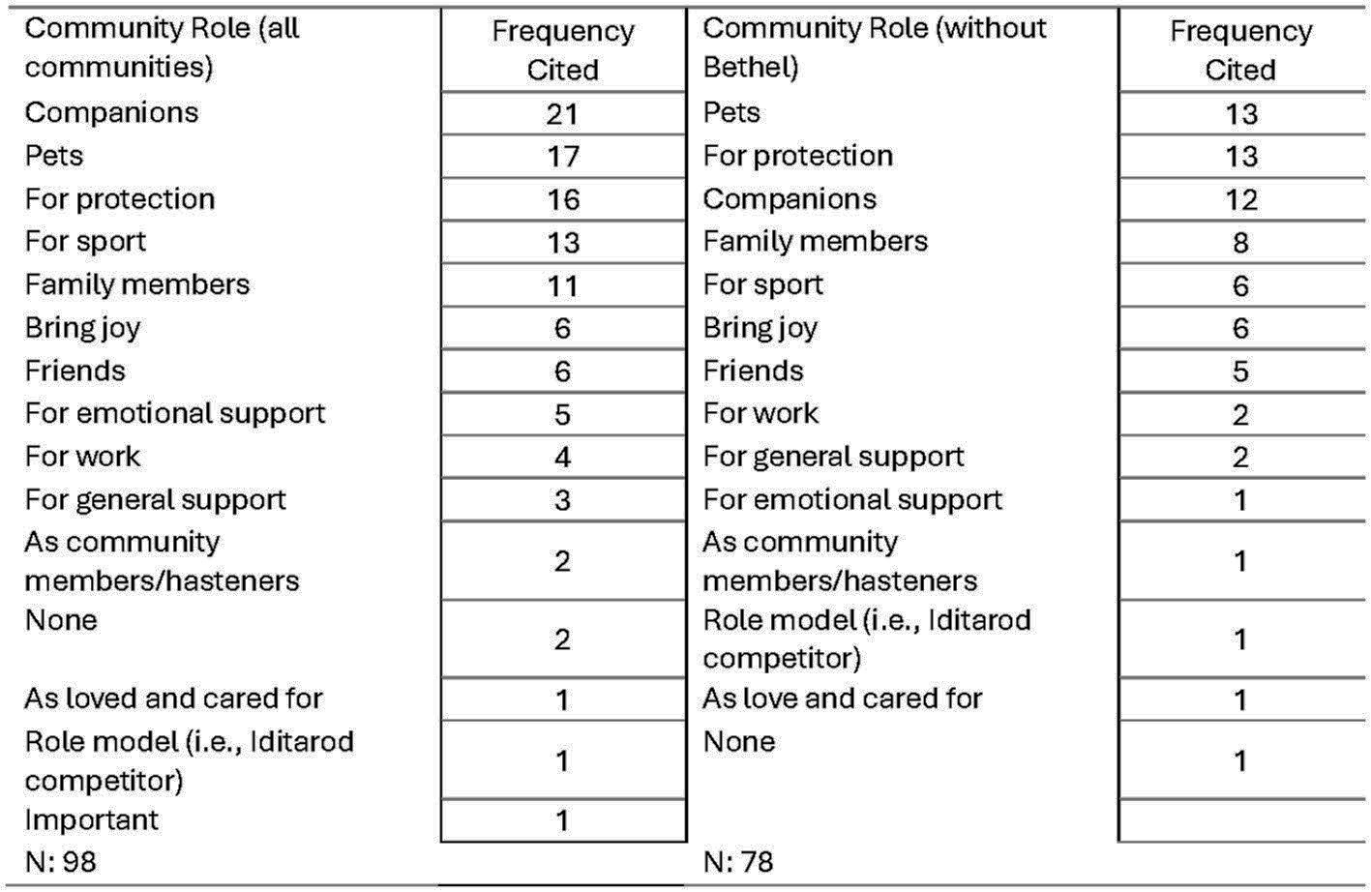
Roles of dogs in communities.

A question with an open text response asked respondents what they believed the role of dogs are in their community. Out of the 98 respondents who provided insights into this, the most frequently noted roles in these rural Alaskan communities were (1) as companions (21%), (2) as pets (17%), and (3) for protection (16%). Dogs are also commonly seen playing a role in sports (13%) and as a part of the family (11%). Fourteen distinct roles were noted when Bethel data is included and thirteen without Bethel (Figure 3); the top three most indicated roles are the same between the data sets. Dogs as “companions” drop from the most noted with Bethel data to the third most noted without Bethel data included.

When asked how respondents feel about dogs, nearly all respondents like dogs (95%) with only one respondent disliking dogs, 1.5% report being afraid of dogs, and 3% reported being indifferent to them. Similarly, when asked about interactions with dogs, most community members have positive interactions with dogs (82%). Only 1% of respondents reported negative interactions while 17% reported neutral interactions. Nearly all respondents have a pet/owner relationship with dogs (93%) rather than a working relationship (2%) or no relationship (5%).

### 3.2 Community perceptions

In sections two, three and four of the survey, respondents were asked for their views on veterinary care, issues that dogs present in the community, and dog related health problems and their costs.

#### 3.2.1 Access to veterinary care

Respondents were asked whether they believe veterinary care is affordable. The majority (54%) were unsure. The remaining respondents were split closely with about 23% believing it is affordable and 24% believing it is not affordable. When removing Bethel data, most respondents were still not sure if veterinary care is affordable, and the proportion increased to 59%.

Respondents were also asked if they felt veterinary care is accessible, with response options of only yes and no available. Results were evenly split with just over half (53%) feeling they can access it. Similarly, 53% of respondents had in fact accessed veterinary care at some point. When Bethel data were removed, results flipped with just under half (48%) feeling they were not able to access care. Again, mimicking perceptions, 48% of non-Bethel respondents report having accessed veterinary care previously.

When expanding on these questions in open text, several themes frequently appear in both the perception of a lack of access (n:52) and being unable to access care in reality (n:17), such as a lack of veterinarians nearby, living in remote areas, cost, and the need to travel to access veterinarians. Within the perception of a lack of access, a sub-theme under the lack of veterinarians was that while some veterinarians travel to the area, but it is infrequent. Supporting this theme, respondents report over 2 years having passed since last seeing a veterinarian when Bethel data were excluded (>27 months). The median time interval between vet visits is similar at 2 years. However, the standard deviation is large (about 22 months) meaning there is significant variation among respondents' answers. It is important to note, as well, that only 39 respondents outside of Bethel answered this question.

#### 3.2.2 Dog-related health risks

Respondents were asked if any of their dogs had been dewormed, vaccinated for rabies, or vaccinated for other diseases. Table 1 illustrates the findings from all communities. Most respondents' dogs were dewormed and vaccinated for rabies. However, this may not be representative of dogs overall in the community due to respondents' likelihood of being clients of the HOP clinics. Most respondents did not have their dogs vaccinated for other diseases, however, and a large portion did not know if their dogs were vaccinated or not.

**Table 1:** Reported rates of deworming and vaccines from all communities.

**Dewormed or vaccinated?**	***N***	**Yes**	**No**	**I don't know**
Dewormed	374	**169 (45%)**	144 (39%)	61 (16%)
Vaccinated for Rabies	366	**226 (62%)**	101 (28%)	39 (11%)
Vaccinated for Other	366	123 (34%)	**137 (37%)**	106 (29%)

Bolded values indicate the largest number.

When Bethel data are removed (Table 2), the majority report not having their dogs dewormed. While the majority are still vaccinated for rabies, the percentage decreases. Similarly, the percentage of dogs vaccinated for other diseases also decreases.

**Table 2:** Reported rates of deworming and vaccines from 24 communities when Bethel removed.

**Dewormed or vaccinated?**	***N***	**Yes**	**No**	**I don't know**
Dewormed	286	112 (39%)	**122 (43%)**	52 (18%)
Vaccinated for Rabies	283	**159 (56%)**	92 (32%)	32 (11%)
Vaccinated for Other	283	75 (27%)	**122 (43%)**	86 (30%)

Bolded values indicate the largest number.

When respondents were asked if they spayed or neutered their dogs, over half (53%) reported spaying or neutering their dogs while 47% reported not doing so. When Bethel data are removed, the percentages change to just under half of respondents reporting spaying or neutering their dogs (48%), and a slight majority did not (52%). Additional insights from respondents into these decisions to spay/neuter their dogs or not were provided. Top cited themes for sterilization included a disinterest in breeding and for population control. Top cited themes for not sterilizing include a lack of access to care, the high cost of care, and the age of the dog.

Respondents were asked how many loose dogs, on average, they see per day in their community. This question was asked with an open-text response option making the analysis complex. For example, many respondents provided ranges (e.g., 5–15 dogs), but others provided ranges without definitive quantities (e.g., 5 or more) and some respondents reported non-quantities such as “lots” or “too many to count.” Therefore, first the quantitative data were transformed into two variables of range minimums and maximums and then assessed in a frequency table (Table 3) with bins increasing by 10 (i.e., 1–10, 11–20, 21–30, etc.). For those ranges without an upper quantitative limit, no data were included.

**Table 3:** Frequency of number of loose dogs reported daily based on qualitative responses.

**Loose dogs reported (qualitative responses)**	**Frequency cited**
Lots	20
Too many	6
More than able to count	6
Many	2
None	1

Overwhelmingly, most respondents report seeing around 10 loose dogs per day (as a minimum, ranges landed between 1–10 dogs per day 254 times, and as a maximum, ranges landed between 1–10 dogs per day 197 times) (Table 4). A smaller but notable portion saw between 10–20 loose dogs per day (as a minimum, ranges landed between 11–20 dogs per day 42 times, and as a maximum, ranges landed between 11–20 dogs per day 27 times). Seeing more than 20 dogs a day was infrequent, and the range from 51 to 90 dogs had no reports. Over 90 was only reported 16 times as a minimum and 15 times as a maximum. However, counts of common qualitative answers expand this picture as there were responses of “too many” and “more than able to count” in many communities.

**Table 4:** Range of loose dogs reported daily.

**Unowned dog reported**	**Minimum**	**Maximum**
1–10	134	104
11–20	18	14
21–30	3	2
31–40	0	0
41–50	2	0
51–60	0	0
61–70	0	0
71–80	0	0
81–90	0	0
91–100	0	0
10–500	1	0

Similar results were found on the question of how many unowned dogs respondents saw per day (Table 5). Also asked with an open text response option (Table 6), many respondents provided quantitative ranges (e.g., 5–15 dogs), though some provided qualitative responses or a mix of the quantitative and qualitative responses. The quantitative data were again transformed into two variables of range minimums and maximums and then assessed in a frequency table. For those ranges without an upper quantitative limit, no data were included. Like loose dogs, most respondents saw around 10 unowned dogs per day (the minimum number of respondents' ranges landed between 1–10 dogs per day 134 times, and the maximum of respondents' range landed between 0–10 dogs per day 104 times). A much smaller but again notable portion of between 11–20 unowned dogs was reported (as a minimum, ranges landed between 11–20 dogs per day 18 times, and as a maximum, ranges landed between 11–20 dogs per day 14 times). Seeing more than 20 dogs was only reported 6 times as a minimum and 2 times as a maximum. Again, as with loose dogs, counts of common qualitative answers reveal that there is an overpopulation of dogs in many areas that respondents couldn't put into numbers.

**Table 5:** Number of unowned dogs reported seeing each day.

**Unowned dog reported**	**Minimum**	**Maximum**
1–10	134	104
11–20	18	14
21–30	3	2
31–40	0	0
41–50	2	0
51–60	0	0
61–70	0	0
71–80	0	0
81–90	0	0
91–100	0	0
101–500	1	0

**Table 6:** Number of unowned dogs reported seeing each day with open text response option using quantitative ranges.

**Unowned dog reported (qualitative responses)**	**Frequency Cited**
Lots	20
None	9
Too many	4
Many	4
More than able to count	3
Hard to know if owned	3
All the time (e.g., Everyday, Every corner on the road, Every hour of the day)	3
Varies on weather	1

These results suggest that loose-owned dogs were more common than loose-unowned dogs as the numbers of loose dogs exceed those of unowned. Respondents were asked how they knew the difference between an owned and unowned dog. Most respondents reported that dogs in their community do not typically have identification (48%), while 28% reported that dogs do have identification and 24% did not know if they do. Themes from respondents' descriptions of how they identify dogs as being owned or unowned are provided include whether the dogs have collars, if they are loose or tied, and if they are visibly cared for.

Questions were asked on respondents' perceptions of the stray, unwanted, and loose dog population. Respondents were asked if stray or unwanted dogs are a problem in their community. Overwhelmingly these dogs are viewed as a problem (71%). Respondents were asked if loose dogs are feared in their community with most indicating they are (69%). Most respondents think dogs spread waste and garbage around their community (75%) while only a few did not (10%). A small portion of respondents weren't sure (15%). This question asked about loose dogs generally, regardless of whether they are owned or unowned.

A reason dogs may be feared (though not exclusively stray or loose dogs) could be due to the prevalence of bites or attacks. As shown in Table 7, equal percentages of respondents think these are a problem as are unsure if dog bites/attacks are a problem (39%) while nearly half this number believe bites/attacks are not a problem (22%). While most respondents have not been bitten by dogs (55%), a substantial portion has been (42%). Not everyone who indicated being bitten in the past answered the follow up question of whether they reported the bite to authorities. Of those who did answer (n:82), most did not report the bite (60%). In total, only 8 respondents gave reasons for reporting, some of whom indicate reporting the bite because they needed medical care at a clinic or hospital. Most of those who did report the bite/attack indicated that their age at the time of being bitten was important (e.g., the respondent was young when bitten). Twenty-nine respondents provided reasons for not reporting, however. The most frequently noted themes from those answers include that little harm was caused by the bite, that the dog was vaccinated, and that the respondent felt it was their fault rather than the dog's.

**Table 7:** Reported prevalence of dog bites/attacks.

**Are dog bites or attacks a problem in your community?**	
**Response**	**Frequency**
Yes	39%
No	22%
I don't know	39%
N: 375	

Respondents were asked to mark all the ways in which the community handles unwanted dogs from a list that included community culls, sending dogs to rescue groups, and owners taking care of it themselves (Table 8). When accounting for all communities, including Bethel, the most common method was scheduled and/or announced community-wide culls (a.k.a. kill days) with 47% of respondents using this method. This was followed by the community sending dogs to rescue groups (45%), and owners directly taking care of unwanted dogs (42%). When removing Bethel data, community cull days remained the most common and increased in frequency (57%), followed by owners directly taking care of unwanted dogs (46%), and lastly the community sending dogs to rescue groups (32%).

**Table 8:** Reported ways in which the community handles unwanted dogs.

**How does your community handle unwanted dogs?**	
Send them out to rescue groups	45%
My community takes care of this (organized, scheduled and/or announced community wide culls (kill days)	47%
Dog owners have to take care of this themselves	42%

N: 345, ^*^Respondents could select more than one response.

Dealing with unwanted dogs can have consequences. In Table 9, most respondents indicated that putting dogs down regularly negatively affected them (50%) while a sizeable portion felt it did not negatively affect them (37%). Some respondents were not sure (13%). When Bethel data were removed, most respondents still indicated that putting down dogs regularly did negatively affect them (46%), but interestingly the portion that felt it did not negatively affect them increased (41%). Those who were not sure stayed the same (14%).

**Table 9:** Consequences of dealing with unwanted dogs.

**If you have to put down dogs regularly does that negatively affect you?**	
**Response**	**Frequency**
Yes	50%
No	37%
I don't know	13%

N:339

### 3.3 Perceived outcomes of HOP

An important aspect of the survey was to assess the impact of the program, HOP, on the communities. First, respondents were asked if and how they had heard about HOP (Table 10). Most respondents had heard of HOP before the program visited their community (61%). Word of mouth was the most common method (53%) followed by Facebook (52%).

**Table 10:** How communities heard of HOP from all surveys.

**How heard of HOP**	**Frequency**
Response	Yes
Hear about HOP before visiting your community?	61%
**Mode of communication** ***(N** = **190)***	
Word of mouth	53%
Facebook	52%
Other (flyer, text message)	19%
Radio	8%

^*^ *Some respondents chose more than one mode*

Next, respondents were asked if they had noticed changes in their community since HOP began (Table 11). Though most respondents were not sure if they had seen a change (51%), of those who had an opinion, the majority thought there were changes in the community since the HOP program began in their community (35%). Only 14% of respondents did not think there had been a change in their community.

**Table 11:** Reported ways in how communities have changed since HOP started.

**Have you noticed any changes in your community since this program started?**	
**Response**	**Frequency**
Yes	35%
No	14%
I don't know	51%

N: 362

**Figure 4:**
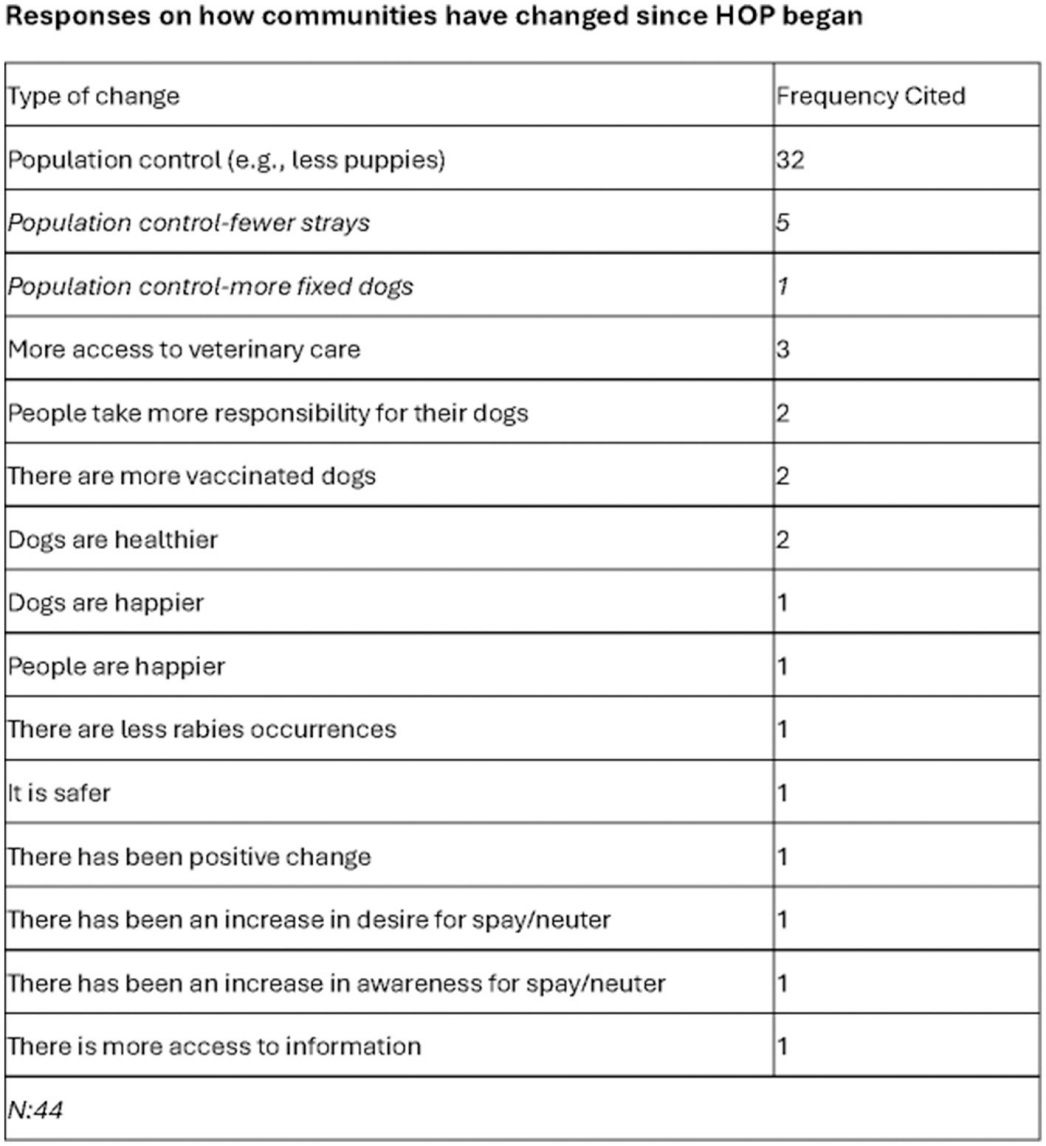
Survey write in responses on how their community has changed since HOP began.

In Figure 4, forty-four respondents gave further insights into how the community has changed. By far the most noted theme was around population control, which was mentioned in 72% of responses. This was brought up in a variety of ways including respondents seeing fewer puppies, fewer strays, and knowing there were more “fixed” or spayed/neutered dogs. Other themes included more access to veterinary care, noticing people taking more responsibility for their dogs, having more vaccinated dogs, and dogs generally being happier and healthier.

Finally, a question was asked if the respondent felt their perspectives of dogs had changed since HOP started in their community. Most respondents were not sure if their view of dogs had changed (51%). Of those who had an opinion, 20% said it did and 29% said it did not. Few respondents expanded on this; only those who said “yes” or “I don't know”. Of those whose views had changed, an increased feeling of safety due to vaccination and liking dogs more were noted themes. Of those who were not sure, one respondent noted that not enough time had passed yet to be able to make a judgement.

## 4 Discussion, conclusion, recommendations and limitations

This study aims to utilize information from the collected surveys to form a basis in literature for veterinary medicine in rural Alaska and to document community perspective input to aid in making veterinary care sustainable in this region. Increasing awareness of the current state of veterinary care and the potential benefits to humans and animals is a key step to improving access to care.

### 4.1 Discussion

From a One Health perspective, it is imperative to understand the role and health of animals for a community to achieve optimum health (14). This is especially true in Alaska Native communities with historical and contemporary ties with dogs (15). The authors found general support for dogs being a part of the community and culture in YK Delta communities. Specifically, nearly all respondents like dogs (95%), 94% of respondents reported owning animals, and dogs are the most common type of animal owned or cared for in this region by far (92%). Most community members report positive interactions with dogs (82%). The role of dogs in these communities, both in villages and in Bethel, is similar; as companions, pets, protection, and sport (i.e., dog mushing) all are in the top roles reported. These findings are comparable with studies completed in the Northwest Territories of Canada, a geographically similar region (6, 16). In this study, data demonstrates that the human-animal bond persists in YK Delta communities despite the cultural shift in roles for dogs, potential disease transmission, and injury risk. This is similar to other studies worldwide (7, 9, 15–17).

When considering risk and community health, it is important to consider contact with animals. An average community surveyed outside of Bethel is ~500 residents with housing units placed close together. And most in-community transportation is via walking (17). Using the survey information collected about the number of loose and unowned dogs seen per day, it is realistic to assume that even non-pet-owning community members will see and interact with dogs daily.

Understanding the affordability and accessibility of veterinary care is important not only for the dog-owning population but also for the public health of the entire community (18, 19). The survey results noted that 54% of respondents are uncertain if veterinary care is affordable and 24% report it is not affordable. The high proportion of respondents being unsure if veterinary care is affordable may be due to never receiving care previously. Further, if they received care under HOP, this was financially subsidized at no cost to the owner, decreasing the interface with veterinary cost structures. When Bethel data was excluded, 59% reported uncertainty and 21% reported it was not affordable. This may be interpreted as the lack of access and exposure to veterinary medicine in remote communities contributing to the uncertainty of affordability.

Like the uncertainty of the affordability of veterinary care reported, a lack of accessibility is reported in over half of survey respondents, with the number one reason provided in frequency being that there is no veterinarian nearby. Other frequent themes included geographic restrictions to accessing care, inconsistency in veterinary teams, and people having to travel to a veterinarian. This aligns with the reality of the veterinary services available in the region: one for-profit veterinarian providing services in Bethel for 5 days a month; the HOP program systematically traveling to YK Delta communities to provide services at no cost to the owners from 2019–2024; and other sporadic visits from non-profit organizations providing care to communities and their pets. Lack of affordable and accessible veterinary care results in the potential for increased zoonotic disease exposure, suboptimal vaccination levels in dogs, and dog overpopulation which contributes to high rates of dog bites (20).

Respondents report only 62% of their dogs have been vaccinated for rabies; when Bethel data is excluded, only a reported 56% of dogs have been vaccinated. These numbers are self-reported by owners, likely by memory and not records, and do not assess vaccination status throughout the 5 years of HOP clinics. This is important to note, as a current rabies vaccine status in the dog is what helps protect the human population from rabies exposure. Based on anecdotal experience the authors expect even lower vaccination rates in the other 33 YK Delta villages that were not visited by HOP; however, this is a hypothesis that requires further investigation.

Rabies is enzootic, or always present at a low level in certain regions in Alaska. Arctic and red foxes living along northern and western coastal Alaska, including the YK Delta, are reservoirs for rabies, however all mammals can be infected with the rabies virus (21). Rabies spillover from foxes to dogs has been known to occur when foxes enter communities seeking food and encounter dogs.

In Alaska, the scope of rabies in these wildlife reservoirs is currently unknown due to the vast land mass and high wildlife populations in the state. Reducing the occurrence of rabies in Alaskan fox populations is not yet feasible. While oral rabies vaccine baits are approved and available for coyotes and racoons, the oral vaccine baits do not work if they are frozen, thus rendering them ineffective much of the time in Alaska (21).

Low rabies vaccination rates in dogs the YK Delta are consistent with the Northwest Territories of Canada where rural community life and geographic challenges are similar. Similar outreach clinics have been used in Canadian communities to provide veterinary medical care that have lacked access. When Calgary-based veterinarians began offering outreach clinics in a handful of remote communities, only 37% of the treated dogs had previously received the rabies vaccine (16). Like the YK Delta, the lack of access to veterinary medical services in these communities is a likely reason for these low vaccination rates. Sustained, continued effort is needed in this region for effective rabies control as the World Health Organization recommends vaccinating at least 70% of dogs in at-risk areas to prevent rabies in humans (22).

There does appear to be a need for increased access to sterilization surgeries in the YK Delta as the surgical sterilization of dogs through spay and neuter surgeries was reported in 53% of respondents, which decreased to 48% when Bethel data was excluded. The literature indicates higher rates are needed. Reece and Chawla (12) found when 65% of the female dogs in a community were sterilized, the dog population declined by 28%(12). Shamshaddini et al. (13) reported a significant reduction in the free-roaming population with 50% annual female dog sterilization. Overall, in the United States, dog surgical sterilization rates have been reported at 69%, and female sterilization is estimated to be 80% of the population (15, 17). The current data may also be subject to sampling bias with surveys distributed during HOP clinics, which did provide surgical sterilization. However, the authors expect even lower surgical sterilization rates in the other 33 YK Delta villages that were not visited by HOP. There appears to be community support for sterilization. Respondents report having their dog spayed/neutered because they were not interested in breeding or having puppies and for population control and the reasons that respondents gave for not having their dog sterilized included barriers rather than a lack of desire.

Loose and stray dogs will reproduce if not sterilized, leading to overpopulation (3, 4, 6, 8, 14, 18). Results indicate that community members do report seeing both loose and unowned dogs daily. However, knowing which dogs are owned can be tricky, with 48% of respondents reporting that dogs do not typically have identification. Respondents instead identify dogs as owned by whether or not the dogs have collars, if they are loose or tied, and if they are visibly cared for. Encouragement of the use of collars for identification is supported by Shurer et al., who saw that the collars brought to clinics for treated dogs became a commodity within communities (3).

Unsurprisingly then, with the high numbers of loose and unowned dogs encountered, community members feel that stray and unwanted dogs are a problem (71%) and loose dogs are feared (69%). Respondents also report that dogs spread waste and garbage around their community (75%). One reason dogs may be feared is the number of bites or attacks that occur. This is consistent with the report of dog bites representing a significant public health threat in Alaska Native children and the recommendation of enhanced animal control and education efforts to reduce dog bite injuries and associated problems with pets and stray dogs (23). Our study found equal numbers of respondents who thought dog bites/attacks are a problem as those unsure if they are (39%). While a majority of respondents have not been bitten by dogs (55%), a substantial amount has been (42%). Most did not report the bite (60%) but few shared details on whether they reported the bite or sought medical treatment. This lack of response could be from feeling indifferent about the bite, not wanting to share personal information, or moving quickly through the survey. Those who did share noted that little harm was caused by the bite, that the dog was vaccinated, and that the respondent felt it was their fault rather than the dog's. Even so, this does not negate the risk of rabies transmission. Importantly, 72% of respondents noticed an improvement in population control in the years that HOP ran clinics in their community.

To control dog overpopulation in these rural communities, difficult decisions must be made. The authors found that the most used methods for handling unwanted dogs in all communities were for the community to organize scheduled and/or announced community-wide culls, for the community to send dogs to rescue groups, and for owners to directly take care of unwanted dogs. This is consistent with findings in Northern Canada, demonstrating the need for consistent care to decrease the population and decrease the prevalence of cull days (8). In addition to the physical health impacts of a lack of accessible and affordable veterinary medicine, the mental health and wellbeing of a community should also be considered. This is especially true in the YK Delta, where there is a negative impact of regularly having to euthanize dogs. This region notably has the highest suicide rates in the entire United States (24). Interesting to note, in response to whether respondents noticed an impact in their communities since HOP started, some respondents said humans and dogs are happier and healthier.

While some might propose that people without the financial means to care for a pet should not have one, literature in this area argues otherwise. Kogan et al. argue that it is not acceptable, nor ethical, to deny families the option to have a pet due to barriers in accessing veterinary health care (5). This is further supported by Wiltzius et al. who suggest that the argument that people with limited means should not have pets is an “untenable solution”(19). Instead, a better understanding of the roles of and attitudes toward animals in these communities is vital to providing better support.

### 4.2 Conclusion

Work on community attitudes toward dogs and access to veterinary care benefits any community-led initiatives, however, published information on veterinary care in rural Alaska is limited. The present study's analysis shows that YK Delta communities want and like dogs despite the associated health concerns. While there are many parallels between remote Northern Canadian communities and Alaskan villages to draw from, gaining nuanced information specific to the region will aid in planning sustainable approaches supported by the communities to improve access to veterinary care.

The present study is the first undertaking to measure this region's community attitudes on these topics. The perspectives gained from this study support the idea that while dogs are a critical and valued aspect of communities, many community members are aware of the problems presented by overpopulation. Specifically, the authors found that personal and community attitudes toward dogs are overwhelmingly positive. Community members are both positively impacted by their relationships with dogs and negatively impacted by the side effects of the overpopulation of loose/stray dogs in communities. The authors also found that perceptions of and actual ability to access veterinary care are low and often cited in responses to other issues. Lastly and in evidence of the low access to care, dogs have low rates of vaccination, and low rates of sterilization, resulting in overpopulation. Consequences of overpopulation noted by respondents included fearing stray and loose dogs, frequency of dog bites and attacks, and negative impacts of having to remove unwanted dogs from the community. The authors hope these results will be used to aid in developing policies, programs, and educational campaigns resulting in improved health outcomes for community members. Next, the authors present recommendations based on the results.

### 4.3 Recommendations

Actions may be taken on the federal, state, city, and village levels to increase accessibility of veterinary care. On the federal level, the United States Department of Agriculture has recognized that there is a shortage of veterinarians in rural areas across the United States and has taken measures to work with states to identify shortage areas and provide incentives to veterinarians to practice in those areas through the Veterinary Medicine Loan Repayment Program (VMLRP). However, this program focuses solely on food animal veterinary medicine and supporting disciplines and does not address the need for veterinary medicine in rural Alaska communities where residents rely on a subsistence lifestyle. Acknowledging the connection between human and animal health and the importance of dogs in rural Alaska at the federal level is critical. Expanding the VMLRP to support veterinarians in the expansion of veterinary services to assist in public health programs would increase veterinary care in Alaska and similar remote locations.

In Alaska, the current statewide services and systems that promote animal and community health are the State Veterinarian and the Lay Vaccinator Program. Alaska has two state public health veterinarians whose duties include disease surveillance, coordinating emergency response in the event of a disease outbreak or natural disaster, coordinating with state and local partners to ensure animal welfare standards are met, and working with food producers to meet requirements for national animal health certification programs. Vaccinating village dogs for rabies provides a buffer between people and wildlife. Since its creation in the 1970s, Alaska's Animal Rabies Lay Vaccinator Program has provided training for people other than veterinarians to administer rabies vaccines to dogs in rural communities (25).

However, even with these statewide services, there continue to be gaps in coverage of rabies vaccination across the state that represent an increased risk for rabies transmission to humans through dog bites. To improve rural veterinary care on the state level, employing or contracting veterinarians to work in each Alaskan region and provide preventative health services, such as those provided by HOP, for disease mitigation should be a consideration. The consistent presence of a veterinarian will work to improve the vaccination and sterilization rates in communities with impacts progressing over time.

At a regional level, in each city or village, initiatives could be launched to identify dogs, a first step to understanding the true concerns and numbers in their village. Groups traveling to the villages could bring collars and tag-making supplies to distribute to owned dogs. It is expected that the dog/human bond will continue to be valued as it has been for centuries, and dogs will continue to be kept in these villages. It takes multiple groups working on the issue of to gather information on the impacts of dogs and community health to inform where veterinary medicine can act. There will always be a place for non-governmental organizations to partner with communities and public health veterinarians.

The information gained from this survey analysis provides insight into small programs and changes that could work toward the goals of improved animal and community health. As such, the authors recommend:

1. A state or federally contracted public health veterinarian to be assigned to each of the major regions in Alaska. Their role would be to provide strategic preventative veterinary medicine services to the remote villages in each region.
2. A system to share information and knowledge between veterinarians and NGOs doing work in villages in Alaska which would inform groups in creating and following a strategy for timing and selection for village visits.
3. When traveling to a village, make time to perform a door-to-door pet census, gathering information on number of pets, date of most recent vaccination, and sterilization status.

a. This information could also be shared in the statewide system.

4. Create a sharable system of rabies vaccination status.
5. Focus on an owned dog identification system through collars and tags.
6. When bringing services to a community, social media and word of mouth are the primary methods of advertising in this region.
7. Specifically understanding the drivers toward care for dogs is helpful when communicating with owners, specifically to encourage sterilization and vaccination, in the YK Delta this driver was prevention of unwanted litters.

Further investigation on additional programs, as well as views from all organizations and stakeholders involved, is suggested. Studies looking at access to veterinary care in other regions of Alaska as well as differing program models and state and federal government roles in public health as it relates to veterinary medicine will help guide long-term solutions.

### 4.4 Limitations

Limitations to this study include survey structure and sampling. Regarding survey structure, the survey was not available in the native regional language of Yupik which may have hindered respondents' comprehension of the questions and ability to respond. Next, the question addressing first-hand experience with a dog bite does not specify the period of when the bite occurred and in future surveys adding this stipulation would allow for the data to be compared to the number of bites reported to YKHC in the same time frame. Lastly, the year the surveys were completed was not tracked. A recommendation for future work in this area is to return to these specific villages and collect data at 3, 5, and 7 years of program engagement.

The data, specifically around affordability and accessibility of veterinary care, is likely subject to sampling bias since HOP clinics were a primary recruiting space. Surveys did not ask whether respondents were HOP clients or if they received subsidized veterinary care nor was this tracked in data entry, so it is unknown how many respondents were HOP clients. Future work should survey villages that both receive regular veterinary care and those that do not.

## Data Availability

The original contributions presented in the study are included in the article/Supplementary material, further inquiries can be directed to the corresponding author.
