# Supplementary material for: Community perspectives on dogs, health risks, and veterinary care impacts in rural Alaska
Source: Front Vet Sci. 2025 Jul 23;12:1602564. doi: 10.3389/fvets.2025.1602564 (PMC12327386; doi:10.3389/fvets.2025.1602564)
Supplement: Supplementary file 1 [file Table_1.docx]

Instructions:

Please answer the questions to the best of your abilities, even if you do not have pets. There are questions on both sides of the paper.

You are not required to answer all survey questions if you feel uncomfortable. Whether you participate or not in this survey will not influence any sort of care you may receive.

1. How do you feel about dogs? (circle one:)
   1. I like dogs
   2. I am indifferent to dogs
   3. I don’t like dogs
   4. I am afraid of dogs
2. How do you interact with dogs? (circle one:)
   1. I own pet dog(s)
   2. I work with dog(s)
   3. I do not interact with dogs
3. My interaction with dogs is (circle one:)
   1. positive
   2. neutral
   3. negative
4. Do you own any animals? (circle one:)

Yes or No

- 1. If yes,
     1. How many animals do you own? ________
     2. What types of animals do you own? Dogs, Cats, Other ______
  2. If no, why do you not own animals? _______

1. What role do you think dogs play in your community? ____________

Veterinary Care Questions:

1. Have you heard of the veterinary profession? (circle one:)
   1. Yes
   2. No
      1. If no, do you know what a veterinarian does? Yes or No
2. Has a veterinarian ever visited your community?

a. Yes

b. No

c. I don’t know

If so, when was the last time a veterinarian visited your community? ________

1. Do you feel you are able to access veterinary care? (circle one:)
   1. Yes
   2. No

If no, why not? ______

1. Have you ever accessed veterinary care for your animal? (circle one:)

a. Yes

b. No

If so, how did you accomplish this?

- - - 1. I called a veterinarian or veterinary technician for advice.
      2. Me and/or my animal traveled outside of my community to visit a veterinarian.
      3. A veterinarian or veterinary group visited my community.
      4. A non-veterinary group helped me access veterinary care.

If you have not accessed veterinary care, why not?______

1. Do you think veterinary care is affordable? (circle one:)
   1. Yes
   2. No
   3. I don’t know
2. Have any of your dogs been dewormed? (circle one:)
   1. Yes
   2. No
   3. I don’t know
3. When was the most recent deworming for any of your dogs? (circle one:)
   1. <1 month ago
   2. < 1 year ago
   3. 1-2 years ago
   4. 3-5 years ago
   5. > 5 years ago
   6. I don’t know
   7. Not applicable
4. Have any of your dogs been vaccinated for Rabies? (circle one:)
   1. Yes
   2. No
   3. I don’t know
5. When was the most recent rabies vaccine for any of your dogs? (circle one:)
   1. <1 month ago
   2. < 1 year ago
   3. 1-2 years ago
   4. 3-5 years ago
   5. > 5 years ago
   6. I don’t know
   7. Not applicable
6. Have any of your dogs been vaccinated for any disease other than rabies? (circle one:)
   1. Yes
   2. No
   3. I don’t know
7. When was the most recent vaccination for a disease other than rabies for any of your dogs?
   1. <1 month ago
   2. < 1 year ago
   3. 1-2 years ago
   4. 3-5 years ago
   5. > 5 years ago
   6. I don’t know
   7. Not applicable
8. If you own a dog or dogs, have you had them spayed or neutered?
   1. Yes
   2. No
      1. If yes, why?
      2. If no, why not?

Community View of Dogs:

1. How do you identify a dog as being owned or un-owned? _____
2. Do dogs typically have identification?
   1. Yes
   2. No
   3. I don’t know
3. Are dogs usually fenced/tied when outdoors?
   1. Yes
   2. No
   3. I don’t know
4. On average, how many loose dogs do you see a day?
5. How many un-owned dogs do you see a day?
6. Are stray or unwanted dogs a problem in your community?
   1. Yes
   2. No
   3. I don’t know
7. Do people in your community fear loose dogs?
   1. Yes
   2. No
   3. I don’t know
8. How safe do you feel being around dogs?
   1. 1= not at all safe
   2. 2 = indifferent
   3. 3 = very safe
9. Are you less willing to exercise outside because of dogs?
   1. Yes
   2. No
   3. I don’t know
10. Do dogs spread waste or garbage around your community?
    1. Yes
    2. No
    3. I don’t know
11. Do you think dogs in your community make people sick?
    1. Yes
    2. No
    3. I don’t know
12. Have you ever been treated for a disease that you may have gotten from your dog?
    1. Yes
    2. No
    3. I don’t know
       1. If yes,
          1. ringworm or other skin problems
          2. GI problems
          3. cysts in your chest or abdomen
          4. other ____________
13. Are dog bites or attacks a problem in your community?
    1. Yes
    2. No
    3. I don’t know
14. If you own a dog, has it ever been bitten by another dog or wild animal?
    1. Yes
    2. No
    3. I don’t know
    4. I don’t own a dog
15. Would you report your if dog had bitten by another dog or wild animal?
    1. Yes
    2. No
    3. I don’t know
       1. Why or why not?
16. Have you ever been bitten by a dog?
    1. Yes
    2. No
    3. I don’t know
       1. If so did you report it?
          1. Why or why not?

This next section will ask questions about the cost of dog related health problems

1. Have you ever received medical treatment for a health issue you experienced from exposure to a dog?

a. Yes

b. No

c. I don’t know

1. Have you ever received medical treatment for a dog bite?
   1. Yes
   2. No
      1. If so, did you have to leave your community to receive treatment? Yes or No
      2. If so, how much money did you have to spend on travel, lodging and meals while away? ___________
   3. Were there medical costs to you that your insurance did not cover? Yes or No
      1. If so, how much?
2. Did you lose wages or the ability to work because of a dog bite?
   1. Yes
   2. No
      1. If so how much money did you lose?
      2. How long were you unable to work or carry out your normal daily activities?
3. How does your community handle unwanted dogs? (You can pick more than one option)
   1. Send them out to rescue groups
   2. My community takes care of this (organized, scheduled and/or announced community wide culls (kill days)
   3. dog owners have to take care of this themselves
4. If you have to put down dogs regularly does that negatively affect you?
   1. Yes
   2. No
   3. I don’t know

Perceived Outcomes

1. Did you hear about this program before it visited your community?
   1. Yes
   2. No
      1. If so, by what means?
         1. radio
         2. facebook
         3. word of mouth
         4. other:
      2. If not, what means of communication would you recommend?
2. Have you noticed any changes in your community since this program started?
   1. Yes
   2. No
   3. I don’t know
      1. If yes, what changes have you noticed?
3. Has your view of dogs in the community changed since this program has started?
   1. Yes
   2. No
   3. I don’t know
      1. Please explain: ___________________
4. Is there anything else you would like to share with us?

Thank you for your participation. Quyana!
